# Perceived value and educational implications of traditional Chinese medicine nursing: a mixed-methods study

**DOI:** 10.3389/fpubh.2026.1763732

**Published:** 2026-02-20

**Authors:** Luxin Wang, Weihong Zhang, Yanlei Wang, Hongxia Wang, Yanyan Han, Xin Wang, Yanfei Liu

**Affiliations:** 1 School of Nursing and Health, Zhengzhou University, Zhengzhou, Henan, China; 2 The Fifth Clinical Medical College of Zhengzhou University, Zhengzhou, Henan, China; 3 Henan Provincial People’s Hospital, Zhengzhou, Henan, China; 4 Henan Province Hospital of TCM, Zhengzhou, Henan, China

**Keywords:** curriculum development, mixed methods, nursing education, perceived value, teaching strategies, traditional Chinese medicine

## Abstract

**Introduction:**

Complementary and alternative medicine is gaining global attention in nursing education, particularly in traditional Chinese medicine (TCM). However, limited research has explored how nursing undergraduates perceive the value of TCM nursing and how their experiences can inform teaching practices. Therefore, this study aimed to investigate nursing students’ perceived value of TCM nursing and to derive educational insights for curriculum improvement.

**Methods:**

A mixed-methods design was adopted. Quantitative data were collected from 172 nursing undergraduates in a Chinese university using a self-developed TCM Perceived Value Questionnaire. Descriptive statistical analyses were used to identify their perceptions and behaviors regarding TCM nursing. Comparisons of the proportion of correct responses between the two universities were performed using the chi-square test or Fisher’s exact test. Qualitative data were obtained through semi-structured interviews with 14 students and analyzed using thematic analysis to explore their perceived value and educational implications for TCM education.

**Results:**

Quantitative findings revealed a generally positive perception of TCM, with 93.6% of participants expressed interest in learning about TCM culture, 77.3% had prior experience with TCM treatments, and 53.3% were aware of national TCM talent policies. Qualitative themes included three key dimensions: (1) perceiving comprehensive impact of TCM nursing values; (2) expecting the effectiveness of TCM learning; (3) optimizing the learning experience of TCM nursing courses.

**Conclusion:**

Undergraduate nursing students demonstrated generally positive perceptions of TCM nursing, recognizing its cultural relevance and clinical potential. However, gaps in knowledge application and instructional delivery remain. To enhance students’ engagement and learning outcomes, TCM nursing education should incorporate experiential teaching methods, strengthen clinical integration, and align content with students’ cognitive, emotional, and behavioral needs. These findings offer practical implications for developing culturally responsive and pedagogically effective TCM nursing curricula.

## 1 Introduction

With the growing global demand for complementary and alternative medicine (CAM), integrating Traditional Chinese medicine (TCM) nursing into modern education has attracted attention for its focus on culturally sensitive, holistic health practices (1, 2). Beyond its cultural significance, TCM represents a comprehensive medical paradigm with distinct diagnostic and therapeutic systems. Cultural identification is prioritized because TCM nursing is anchored in holistic health philosophies that differ from the reductionist model of Western medicine (3). In this context, cultural identification is not merely an appreciation of tradition but the cognitive foundation necessary for students to internalize TCM’s medical logic and apply its clinical principles effectively. In clinical practice, TCM nursing requires consideration of a wide range of internal and external factors, such as body constitution, psychological state, and social and environmental influences—which are regarded as essential to promoting overall health and well-being (2, 4). Ultimately, TCM instruction plays a vital role in preparing culturally competent, well-rounded nursing professionals by enhancing both their professional competence and awareness of holistic care (5).

The ‘Action Plan for Further Improving Nursing Services (2023–2025)’ (6), jointly promulgated by the National Health Commission and the National Administration of Traditional Chinese Medicine in June 2023, explicitly emphasizes the necessity of enhancing TCM nursing capabilities and standardizing the training of TCM nursing professionals. This policy aims to develop a specialized health workforce capable of addressing the complex needs of an aging society and chronic disease management through integrated East–West nursing practices. In alignment with these national strategies, nursing colleges have developed structured TCM curricula. The scope of instruction typically spans from TCM Basic Theories to clinical techniques such as acupressure and moxibustion. The educational objective is graduated: moving from cultivating ‘cultural awareness’ in early years to developing ‘clinical application competence’—during senior clerkships (7). Graduates are expected to integrate TCM nursing’s holistic perspective into patient-centered care plans.

However, students often perceive TCM courses as abstract and leading to low engagement and limited skill development (8). While students generally recognize the therapeutic value of TCM techniques during clinical placements, they frequently encounter barriers to implementation, such as institutional restrictions, lack of clinical guidance, and patient skepticism (9, 10). Therefore, gaining a comprehensive understanding of students’ perceptions and learning experiences in TCM nursing is essential for optimizing curriculum content and advancing nursing talent development (7). However, the extent to which nursing students understand, accept, and apply TCM principles remains underexplored. Existing research has largely focused on curriculum structure or faculty perspectives, with limited attention paid to students’ experiences and perceptions.

Students’ views on TCM nursing are not only influenced by the course content, but also cognition, emotion, and behavior. Studies showed while nursing students may respect indigenous or complementary therapies, they often lack formal exposure or perceive such methods as lacking scientific rigor (11, 12). These attitudes mirror some Chinese students’ ambivalence toward TCM nursing—recognizing its cultural significance but questioning its clinical applicability (13, 14). This suggests that students’ perceptions are shaped not only by curricular content but also by underlying theories of perceived value and cultural competence, which emphasize the cognitive, emotional, and behavioral dimensions of how individuals appraise and engage with culturally embedded practices (15–17). Despite these dynamics, few studies have systematically explored nursing students’ perceived value of TCM nursing, particularly from a mixed-method perspective that combines qualitative depth with quantitative breadth. While research has focused on educators’ views, clinical nurses’ experiences, or patients’ attitudes (18–20), there remains a lack of systematic investigation into how undergraduate nursing students perceive its value. Yet students’ voices are essential for informing the development of culturally responsive and engaging TCM nursing curricula.

Therefore, this study adopts an explanatory sequential mixed-methods approach to investigate nursing undergraduates’ perceptions, experiences, and practice behaviors toward TCM nursing through the lens of perceived value theory. By combining individual interviews and a cross-sectional survey, we aim to identify barriers, facilitators, and educational implications that can inform evidence-based curriculum reforms and improve the cultural competence of future nurses.

## 2 Methods

### 2.1 Study design

**Figure 1:**
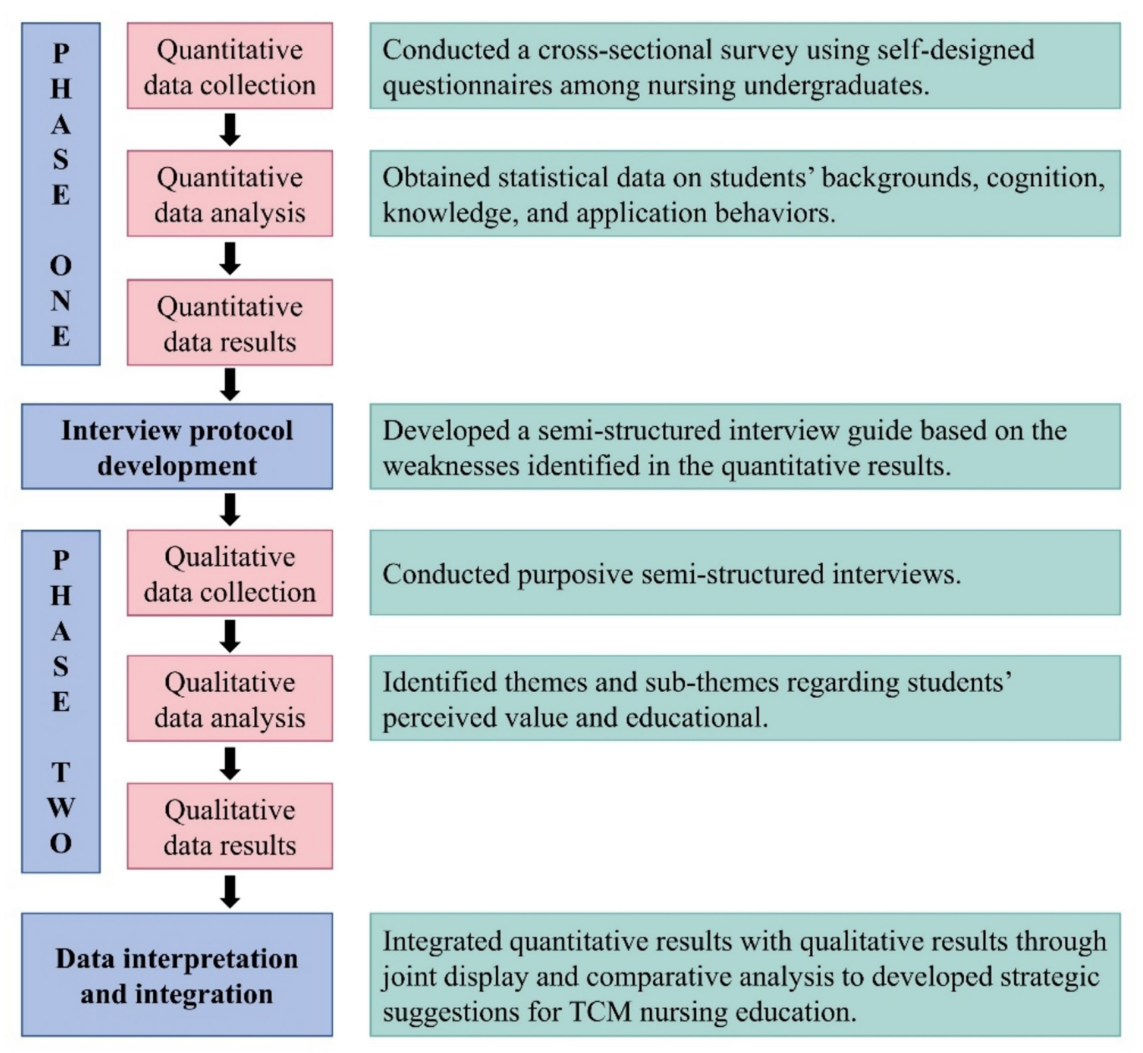
Procedure for the explanatory sequential mixed-methods design.

This study employed an explanatory sequential mixed-methods design to explore nursing undergraduates’ perceived value, experiences, and behavioral intentions regarding TCM nursing (Figure 1). Firstly, quantitative data were gathered using a questionnaire. Subsequently, based on the results of quantitative analysis, in-depth interviews were conducted with students to further explain the preliminary quantitative findings (21). The study followed the Guidelines for Reporting on Mixed Methods Studies (GRAMMS) (22) and was approved by the Ethics Committee of Zhengzhou University Life Sciences (Approval No.: ZZUIRB 2025-01). The conceptual framework was informed by Perceived Value Theory and Cultural Competence Theory, which supported the investigation of cultural beliefs and educational experiences in TCM nursing education.

### 2.2 Participants and setting

#### 2.2.1 Quantitative study

The target population included nursing undergraduates enrolled in a full-time four-year program across two universities in Henan Province. Participants who had never taken a TCM nursing course or declined to participate were excluded. The two institutions represent the two dominant systems of nursing education in China: a TCM-focused model and a Western medical model. Both are located in the same region and hold equivalent academic status, thereby minimizing potential confounding effects of regional economic disparities. The TCM-oriented university offers nursing students TCM and TCM-nursing required courses (Introduction to TCM, TCM Classics, Fundamentals of TCM Nursing, Clinical TCM Nursing), together with electives and skills-lab sessions. The Western-medicine-oriented university provides only one TCM-related course—TCM Nursing—covering basic TCM nursing knowledge, skills-lab training, and clinical observation.

The cross-sectional survey was conducted using convenience sampling between November 2024 and December 2024. Variables included demographic data, TCM cultural background, cognition of TCM, behavior of applying TCM, and understanding of TCM-related policies. Based on the sample size estimation method proposed by Ni et al. (23), and accounting for a 20% attrition rate, a minimum of 113 participants was required. Yet 172 nursing undergraduates completed the survey.

#### 2.2.2 Qualitative study

In January 2025, purposive sampling was used to recruit 14 participants for semi-structured interviews, ensuring diversity in gender, academic year, and exposure to TCM. Interviews continued until data saturation was reached, defined as the point when no new codes or categories emerged relevant to the research questions (24). Participants were coded from A1 to A14. Notably, these interviewees were exclusively recruited from the Western medicine-oriented university. This sampling strategy was a deliberate decision guided by the preliminary quantitative results, which revealed that students from the Western medicine-oriented institution exhibited significantly lower scores in the cognitive and behavioral dimensions of perceived value compared to their counterparts in the TCM-oriented institution. By focusing on this specific cohort, the qualitative inquiry aimed to explore the roots of their relative disengagement and identify the unique barriers they encounter.

### 2.3 Data collection

The survey instrument was developed by integrating items from multiple published studies (15, 25–31) to ensure comprehensive coverage of the research domains. To ensure content validity, the initial draft was reviewed by a panel of experts (e.g., TCM nursing specialists and educators) to confirm its clinical and cultural relevance. A pilot test was subsequently conducted with 15 nursing undergraduates to assess the clarity and readability of the questions; minor adjustments were made to the phrasing based on their feedback. The structured questionnaire consists of five main sections: (1) general demographics; (2) TCM cultural background; (3) cognition of TCM nursing; (4) knowledge of TCM nursing; (5) behavior of applying TCM nursing; (6) awareness of TCM-related policies (see Appendix A). The survey was delivered both online and offline. Trained research assistants provided consistent instructions. Questionnaires were reviewed and validated on-site to reduce missing data.

Interviews were employed face-to-face in private classrooms after obtaining informed consent, and conducted by the first author. Main questions were regarding their view on TCM nursing, perceptions, experiences, and practice behaviors. Each interview lasted 30–40 min, guided by the perceived-value framework, followed an interview protocol covering five domains: (1) perceptions of TCM nursing; (2) learning experiences and emotional responses; (3) CHANGES in cultural understanding and attitudes; (4) impact on personal life, family, and academic growth; (5) suggestions for improving TCM nursing education. The specific interview outline was shown in Appendix B.

### 2.4 Data analysis

Quantitative data were processed using SPSS 25.0 for descriptive statistics and consistency checks. Comparisons of the proportion of correct responses between the two universities were performed using the chi-square test or Fisher’s exact test. Data were independently entered by two coders and cross-verified to ensure accuracy. The significance level for all tests was set at *p*-value <0.05.

Interviews were audio-recorded, supplemented with notes, and transcribed verbatim within 24 h. The researchers performed a comprehensive thematic immersion by repeatedly reviewing the data and notes, analyzing participants’ behavioral and emotional expressions. Colaizzi’s (32) seven-step phenomenological approach was used to analyze qualitative data, including: (1) familiarization: The researchers carefully read all the data; (2) extraction of significant statements; (3) formulation the researchers formulated/coded meanings for recurring viewpoints; (4) clustering of coded viewpoints; (5) writing a detailed and comprehensive description with no omissions; (6) identification of similar viewpoints; (7) verification through return visits to the participants. Two researchers who had undergone training in qualitative research courses and forums performed data coding, acquiring expertise in qualitative research methods and interview skills, coupled with experience of leading qualitative research interviews. The study employed investigator triangulation with independent coding to minimize bias (33). Any coding discrepancies were resolved through team consensus.

Data integration and interpretation were achieved through the construction of a joint display, aimed at generating meta-inferences through cross-validation and complementary elaboration of the findings. Following a contiguous approach, the quantitative and qualitative results are presented side-by-side within the text to facilitate a synergistic weaving of data (34). During this integrative stage, both datasets were systematically merged to assess for confirmation, expansion, or discordance. Confirmation was identified when the qualitative insights reinforced the statistical trends, leading to convergent conclusions (35). Expansion occurred when the qualitative narratives further elucidated the quantitative results by providing additional, non-overlapping interpretations that enriched the overall findings. Conversely, any discordance—where quantitative and qualitative results diverged or led to conflicting interpretations—was critically examined to reveal underlying complexities. This systemic integration provides a comprehensive explanation for the participants’ cognitive characteristics, the underlying factors, and the resulting educational implications.

## 3 Results

### 3.1 Quantitative findings

#### 3.1.1 Participant characteristics

Of the 180 distributed questionnaires, 172 were returned (response rate: 95.6%), with 8 excluded due to incompleteness. The demographic characteristics are shown in Table 1. 56.4% were from Western-medicine-oriented university, and 43.6% from TCM-oriented university. A majority of participants were female (80.8%). Most were in their junior year (64.0%). There were no statistically significant differences in gender and grade distribution between students from the two universities.

**Table 1:** The characteristics of quantitative study participants from the different universities.

Variable	All universities (*N* = 172, %)	Western-medicine-oriented university (*n* = 97, %)	TCM-oriented university (*n* = 75, %)	*X^2^*	*p*-value
Gender				0.294	0.587
Female	139 (80.8)	77 (79.4)	62 (82.7)		
Male	33 (19.2)	20 (20.6)	13 (17.3)		
Grade				0.177	0.981
Freshman year	14 (8.1)	8 (8.2)	6 (8.0)		
Sophomore year	16 (9.3)	9 (9.3)	7 (9.3)		
Junior year	110 (64.0)	63 (64.9)	47 (62.7)		
Senior year	32 (18.6)	17 (17.5)	15 (10.0)		

#### 3.1.2 Exposure to TCM nursing

Among the participants, 142 students (82.6%) had received formal instruction in TCM nursing courses. Most students were unfamiliar with the specific content related to TCM cultural elements as outlined in the China Citizen Scientific Literacy Benchmark. 113 students (65.7%) had attended lectures or seminars on TCM knowledge and culture.108 students (62.8%) reported that their schools had organized TCM-themed science popularization events. Participants reported learning about TCM primarily through the Internet (77.9%), school courses (75.6%), books and newspapers (57.0%), and television programs (53.3%). Over two-thirds of participants had read TCM-related books or literature. Among them, 136 (79.1%) reviewed by reading textbooks and notes, 108 (62.8%) watched online video courses, 103 (59.9%) completed practice questions, and 77 (44.8%) discussed content with classmates. Only 7 students (4.1%) reported that they never reviewed the after class. For the items “Understood the content of the China Citizen Science Literacy Benchmark,” “Attended a lecture about TCM,” and “Relatives work in the field of TCM,” no significant differences were observed between the two universities (*p* > 0.05). Conversely, statistically significant disparities were identified in “Lectures held on TCM culture in school” and “Read books about TCM” (*p* < 0.05). Further statistical data are shown in Appendix C Table 1.

#### 3.1.3 Attitudes and cognition of TCM nursing

Regarding attitudes and cognition of TCM nursing, Appendix C Table 2 shows that the response patterns for each items differed significantly between the two groups; no significant differences were found for the remaining items. The majority of nursing undergraduates expressed strong endorsement of TCM nursing, as evidenced by their agreement to offer TCM nursing courses (93.0%), their willingness to learn TCM nursing (93.6%), and their recognition of its effectiveness (89.5%).

#### 3.1.4 Knowledge and understanding of TCM nursing

Appendix C Table 3 presents the varied correct response rates to knowledge-based questions. While 85.5% correctly identified the concept of the “Five Viscera” in TCM, and 93.0% selected the correct container for decocting herbal medicine, fewer students demonstrated understanding of more abstract cultural concepts, such as the “Four Great Classical Works of TCM” (20.3%) and the “Core Spirits of TCM Culture” (30.8%). In total, 57.0% correctly answered questions regarding basic TCM theory, and 64.5% understood the Five Elements generation cycle. Significant between-group differences were found between the two universities for “The generative cycle of the Five Elements” and “The effects of TCM nursing.”

#### 3.1.5 Acceptance and application of TCM nursing practices

More than half (51.2%) of the students could correctly identify the therapeutic effects of TCM. A total of 77.3% of the students had received TCM treatments, with herbal prescriptions and acupoint massage being the most common approaches. Following the completion of TCM nursing courses, students reported increased willingness to apply TCM wellness principles to daily life. Specifically, 47.7% expressed that they would voluntarily practice Baduanjin to maintain physical health. Despite this, 32.6% of students were unwilling to purchase TCM-related health products, and 22.1% expressed reluctance to adopt TCM-based health maintenance methods. Encouragingly, 80.8% stated they would promote TCM wellness knowledge to their family and friends, and 96.5% believed that the promotion and inheritance of TCM culture is necessary. Among these items, “Modes of seeking medical treatment,” “Purchased products related to TCM health care” and “The actively adopted TCM health methods” showed a statistically significant difference, while other items showed no statistically significant difference (see Appendix C Table 4).

#### 3.1.6 Awareness of national TCM-related policies

Awareness of national-level TCM policies was relatively limited among the nursing undergraduates. Approximately 53.3% were aware of government strategies to support and cultivate TCM talents, while about one-quarter were completely unfamiliar with such policies. Regarding knowledge of medical insurance coverage for TCM treatments, 39.0% of students reported being completely unaware, while only 27.9% had a better understanding of the related insurance policies (see Appendix C Table 5).

### 3.2 Qualitative findings

**Figure 2:**
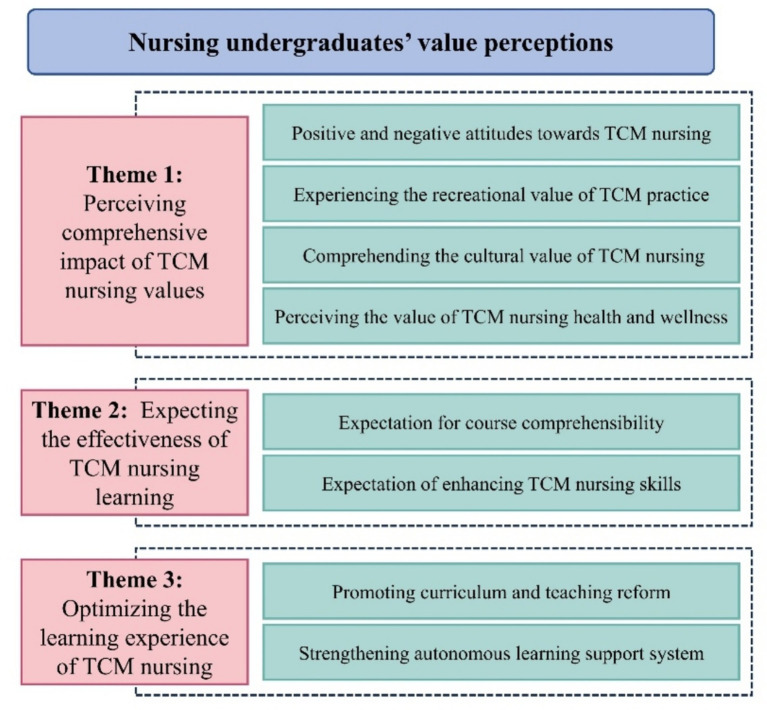
The main themes and sub themes of the qualitative study.

Initial quantitative results indicated that nursing undergraduates from both TCM and Western medicine universities maintained high levels of recognition and positive attitudes toward TCM nursing. However, Western medicine students exhibited significant deficits in TCM knowledge, clinical application, and awareness of relevant policies. To further elucidate the factors contributing to this phenomenon and to explore specific barriers within their educational environment, a subsequent qualitative phase was conducted. A total of 14 undergraduate nursing students from a Western-medicine-oriented university participated in semi-structured interviews. Among them, 12 were female, and 10 were in their junior year. Detailed demographic information is presented in Table 2. The qualitative study identified three major themes and eight sub-themes, as illustrated in Figure 2.

**Table 2:** The characteristics of qualitative study participants.

Variable	All participants (*n* = 14, %)
Gender	
Female	12 (85.7)
Male	2 (14.3)
Grade	
Junior year	10 (71.4)
Senior year	4 (28.6)

#### 3.2.1 Theme 1: perceiving comprehensive impact of TCM nursing values

This theme reflects participants’ cognitive, emotional, and behavioral responses to the learning experience of TCM nursing, as well as its perceived cultural and health-related significance.

##### 3.2.1.1 Sub-theme 1: positive and negative attitudes towards TCM nursing

12 participants reported gaining a structured understanding of TCM nursing and its theoretical underpinnings. A7 remarked: “I gained a systematic understanding of TCM. We mainly learned about its vastness—it wasn’t in-depth, but I now have a general idea of the system.” A13 mentioned: “TCM nursing is an integral component of Chinese medicine, synthesizing traditional theories with clinical practices. By emphasizing a holistic perspective, it serves as a highly valuable nursing paradigm.”

However, several students expressed limited interest, regarding the content as exam-oriented and disconnected from their daily lives. A14 stated: “Once the exam is over, I forget everything we learned in class. Whether I take this course [TCM nursing] or not has very little impact on me.” A14 showed: “I also gave acupuncture a try, but honestly, I do not think it has much therapeutic value. The effects felt a bit like a gimmick.”

##### 3.2.1.2 Sub-theme 2: experiencing the recreational value of TCM nursing practice

Practical experiences such as lab demonstrations and experiential learning enhanced students’ interest. A5 shared: “What impressed me were the lab classes—acupuncture, Baduanjin exercises—those were really engaging.” A8 expressed: “What impressed me most were the practical sessions. During my clinical clerkship at the TCM hospital, I observed several TCM procedures: moxibustion, cupping, and acupuncture. I noticed that needles vary in length and shape, and targeting different acupoints leads to different therapeutic effects. I found it quite fascinating.”

##### 3.2.1.3 Sub-theme 3: comprehending the cultural value of TCM nursing

Exposure to TCM nursing improved students’ recognition of its cultural depth and scientific foundation. A1 shared: “Previously, I might have regarded TCM as something quite mystical and mysterious—a form of healing that can only be felt but not expressed in words. However, after taking this course, I realized that, just like Western medicine, it is supported by a vast and reliable theoretical framework.” A12 noted: “My understanding has changed significantly. It now feels systematic and carries deep cultural meaning.”

##### 3.2.1.4 Sub-theme 4: perceiving the value of TCM nursing health and wellness

Several students reported applying learned TCM techniques to self-care and preventive health. A1 commented: “With this knowledge, I can treat minor ailments myself and avoid unnecessary trouble.” A8 highlighted: “I personally belong to the Yin-deficiency and internal heat type; my symptoms are quite classic. My best friend, on the other hand, is a typical case of Spleen-deficiency and Yang-deficiency. Based on these classifications, we now pay more attention to our daily diets and herbal teas to maintain our health.”

#### 3.2.2 Theme 2: expecting the effectiveness of TCM learning

This theme captures students’ aspirations regarding the course’s academic utility and practical applicability.

##### 3.2.2.1 Sub-theme 1: expectation for course comprehensibility

7 participants described the course content as abstract and challenging. A9 stated: “Even though the teachers explained things in relatively simple terms, it was still quite difficult to learn because we lacked a foundation in TCM knowledge. My overall feeling was that the content was somewhat obscure; there were even certain characters that I did not recognize at all.” Given the perceived complexity of the curriculum, many students expressed a strong desire for enhanced instructional comprehensibility. A7 expressed: “I hope teachers can simplify the content and use visual aids or clinical cases to make it more digestible.”

##### 3.2.2.2 Sub-theme 2: expectation of enhancing TCM nursing clinical skills

Students expressed a desire to apply TCM nursing techniques in everyday life and promote them to family and friends. A2 stated: “I hope to practice techniques like gua sha and cupping on my family to relieve discomfort.” Conversely, a few found it impractical in daily life. A11 admitted: “I just study this to pass exams. It does not seem very useful to me.” A12 said: “I also gave acupuncture a try, but honestly, I do not think it has much therapeutic value, and I find it rather boring. The effects felt a bit like a gimmick.”

#### 3.2.3 Theme 3: optimizing the learning experience of TCM nursing courses

Students provided constructive suggestions based on their learning challenges and classroom experiences.

##### 3.2.3.1 Sub-theme 1: promoting curriculum and teaching reform

Participants advocated for more engaging and clinically relevant content. A5 described: “I feel that practical sessions are quite important. Looking back, what impressed me most were the lab classes, where the teacher showed us the actual equipment and materials while explaining the theory. Seeing the tools in person while hearing the explanations was incredibly helpful for my understanding of various TCM nursing procedures.” A13 suggested: “We need more hands-on practice and integration of modern medicine explanations into TCM theories.”

##### 3.2.3.2 Sub-theme 2: strengthening autonomous learning support system

Faced with complex content, students proactively sought alternative learning strategies. A4 shared: “I often turn to teachers, classmates, and online resources for help.”A13 humorously added: “Sometimes I ask Kimi (AI assistant), but it does not always give the answer I need.”

### 3.3 Mixed method results

The qualitative findings provided a complementary perspective, offering a nuanced validation of the prior quantitative results. As summarized in the joint display (Table 3), the merging of these datasets facilitates a deeper understanding of the findings, where qualitative narratives elucidate the statistical patterns observed in the initial phase.

**Table 3:** Joint display of quantitative and qualitative results.

Quantitative findings	Qualitative findings	Integrated insights
*Positive attitude of TCM nursing:* The majority of nursing undergraduates expressed strong endorsement of TCM nursing, as evidenced by their agreement to offer TCM nursing courses (93.0%), their willingness to learn TCM nursing (93.6%), and their recognition of its effectiveness (89.5%).	*Positive and negative attitudes towards TCM nursing:* 12 participants reported gaining a structured understanding of TCM nursing and its theoretical underpinnings. However, several students expressed limited interest, regarding the content as exam-oriented and disconnected from their daily lives.	Both datasets are highly congruent. Nursing undergraduates have developed a deep emotional resonance and value alignment with TCM nursing.
*Knowledge and understanding of TCM nursing:* Few students demonstrated understanding of more abstract cultural concepts.	*Expectation for course comprehensibility:* 7 participants described the course content as abstract and challenging. Given the perceived complexity of the curriculum, many students expressed a strong desire for enhanced instructional comprehensibility.	Qualitative insights explain the underlying quantitative results. The low level of knowledge is not merely a result of memory failure but stem from a “linguistic barrier” between Western and TCM paradigms. And students expect some teaching reform to enhance course comprehensibility and TCM nursing clinical skills.
*Polarized application behaviors:* Some students were eager to apply TCM nursing practices, while others refrained entirely from using them.	*Perceiving the value of TCM nursing health and wellness:* Several students reported applying learned TCM techniques to self-care and preventive health. Students have various levels of belief in TCM’s “scientificity.”	Qualitative insights explain the underlying quantitative results. The divergence in behavior is rooted in varying levels of belief in TCM’s “scientificity.” A demand for empirical evidence dictates the transition from knowledge to practice.

## 4 Discussion

### 4.1 Nursing students have high cultural identification with TCM but lack practice behaviors

Qualitative interviews revealed a profound interest in TCM nursing among students, which is highly congruent with the quantitative findings showing that 93.6% of participants expressed a willingness to learn and 89.5% endorsed its therapeutic efficacy. Paradoxically, this strong cultural identification has not effectively translated into practical behaviors. Quantitative data indicated that 32.6% of students had never purchased TCM nursing healthcare products, providing empirical evidence that aligns with the ‘limited clinical applicability’ frequently mentioned by students during the interviews. According to the Perceived Value Theory, individual behavioral choices depend on the subjective assessment of benefits and costs (36). Although students have a high perception of the emotional and cultural values of TCM culture, their behavioral willingness decreases when the actual benefits perceived during practice (e.g., clinical application effects, patient benefits) are insufficient, or when the perceived costs of learning and application (e.g., difficulty of TCM theories, disconnection between the classroom and the clinic, etc.) are too high. On the other hand, Cultural Competence Theory emphasizes the need for nursing staff to build intercultural nursing competence in multiple dimensions such as awareness, knowledge, skills, practical experience and intrinsic motivation (37). Ingram (38) states that cultural competence involves not only knowledge of multicultural values, beliefs, and practices, but also learning reinforced by actual exposure and practice. In this study, the nursing undergraduates had a strong identification with TCM culture, which indicates that they have a foundation of ‘cultural awareness’, but their cultural competence is still incomplete without sufficient exposure to and training in TCM practice (37). It suggests that providing culturally sensitive care in multicultural environments is the key to reducing health inequalities and improving the quality of care (37), and cultural competence is not enough to meet the requirements of nursing care if it is only a matter of identification but not actual action.

### 4.2 Reasons for high cultural identity in TCM and educational strategies

The phenomenon of students’ strong cultural identification with TCM but weak behavioral transformation may be caused by the following reasons: firstly, the content and structure of teaching are single. At present, many nursing colleges and universities treat TCM nursing as a separate elective or theoretical course, the curriculum lacks systematicity and depth, and theory and clinical practice are separated, making it difficult for students to apply what they have learnt in real-life situations (39). Secondly, knowledge translation barriers. The abstract concepts of TCM theories have a high threshold of understanding for nursing undergraduates, coupled with the fact that traditional TCM terminology is not the same as the language of modern medicine, which makes it difficult for students to flexibly apply what they have learnt about TCM to modern nursing practice. This quantitative evidence is further corroborated by the qualitative theme: while students demonstrated a high level of identification with fundamental concepts such as Yin-Yang and the Five Elements, a significantly smaller proportion possessed a deep understanding of more abstract cultural constructs. This cognitive discrepancy elucidates the sense of frustration regarding theoretical comprehension expressed by students during interviews, highlighting a critical gap between cultural identification and actual knowledge mastery. The lack of case studies and practice opportunities also prevents students from experiencing the actual efficacy of TCM nursing, thus reducing learning motivation (40). Finally, insufficient system support. The lack of platforms or incentives for TCM nursing practice in school and clinical settings, such as dedicated TCM nursing training bases, internship opportunities in TCM hospitals, and instructors, greatly limits students’ ability to translate classroom knowledge into nursing behaviors (41). Additionally, there is a lack of uniform clinical pathways and evidence-based guidelines for TCM’s nursing, leading students to be skeptical about the efficacy of TCM nursing (42, 43).

### 4.3 Teaching implications of TCM nursing education

The concepts and knowledge of TCM nursing are integrated into the nursing curriculum to optimize the curriculum system and achieve the integration of theory and clinical practice. For example, combining TCM nursing cases with teaching in professional courses such as internal surgery, obstetrics and gynecology (44), using experiential teaching methods such as case discussion and scenario simulation can enhance students’ practical perceptions (45, 46). Lin et al. (39) demonstrated that a 32-credit hour TCM foundation course significantly enhanced students’ knowledge of TCM and willingness to integrate treatments, suggesting that a more systematic teaching program for TCM nursing.

Furthermore, by reinforcing the clinical evidence of TCM nursing and the analysis of medical records, students can intuitively feel the positive effects of TCM methods on patients’ health and enhance their perceived value of TCM (47). Experienced TCM nursing experts can be invited to give special lectures or demonstrations to show successful cases of TCM nursing and help students recognize the unique advantages of TCM nursing in holistic health management, thereby increasing their recognition of TCM’s practical value.

Additionally, focus on the development of students’ “Awareness-Knowledge-Skill-Encounter-Desire” is important to improve students’ cultural competence according to the Campinha-Bacote’s process of cultural competence model (48). For example, experiential activities such as role-playing, teacher-student exchange visits and cross-cultural exchanges are introduced to enable students to understand multicultural perspectives and develop cross-cultural communication skills and service awareness in real-life exchanges. Studies (49) have pointed out that multicultural curricula and practical activities can effectively enhance nursing students’ intercultural nursing competence.

Finally, to strengthen systematic support, schools should co-operate with TCM organizations to establish TCM nursing practice bases to provide authentic clinical observation and operation opportunities (50). Teaching teams with both Chinese and Western medicine backgrounds should be trained, and TCM nursing syllabi and guidelines should be compiled to form institutional support (47). Emphasis has also been placed on integrating traditional medicine education internationally, the World Health Organization has recognized the value of TCM by incorporating TCM diagnostic codes into the International Classification of Diseases in 2019 (51), and tertiary nursing education should also follow this trend and improve the corresponding educational policies and resource support.

### 4.4 Limitations

This study still has some limitations. First, the single-institution design in Henan province may limit generalizability due to regional particularities in both cultural attitudes toward TCM and curriculum implementation. For instance, students with more than 3 years of residence in inland China demonstrated stronger confidence in TCM nursing development and a higher willingness to participate in TCM training programs. Second, the sample was confined to undergraduate nursing students through convenience sampling, which may introduce selection bias and limit the comprehensiveness and generalizability of the findings, as students with a pre-existing interest in TCM might have been more likely to participate. Third, social desirability bias cannot be entirely ruled out. Participants might have provided responses they perceived as professionally ‘correct’ or favorable to the researchers, particularly regarding the significance of TCM nursing, which may not fully reflect their private clinical skepticism. To effectively enhance the teaching quality of TCM nursing courses and to achieve the anticipated goal of cultivating nursing professionals with a high-quality international perspective, future nursing education needs to focus on how to better align teaching practices with the overarching objectives of nursing talent development.

## 5 Conclusion

Nursing undergraduates’ perceived value and knowledge of TCM nursing are pivotal to the advancement of TCM nursing education and the enhancement of teaching quality. This study found that nursing students recognize the cognitive value, recreational value, cultural value, and health and wellness value of TCM nursing. Notably, 93.6% of respondents expressed a willingness to learn about TCM culture, reflecting a strong emotional and cultural acceptance. However, this cultural identity has not fully translated into practice. Students’ perceived practical value of TCM nursing remains limited, and many reported difficulties in understanding the curriculum due to its abstract theoretical foundations and divergence from Western medical paradigms. These findings suggest a gap between students’ cultural recognition of TCM and their behavioral willingness or ability to apply TCM nursing in clinical settings. To bridge this gap, future curriculum design should be guided by theoretical frameworks such as Perceived Value Theory and Cultural Competence Theory, emphasizing the integration of affective, cognitive, and behavioral dimensions of learning. A more robust, interdisciplinary educational model is needed—one that synthesizes TCM with evidence-based modern nursing, enhances practical teaching, and incorporates real-world clinical case studies and simulations to deepen students’ trust in and understanding of TCM nursing.

## Data Availability

The data analyzed in this study is subject to the following licenses/restrictions: the data that support the findings of this study are available on request from the corresponding author. Requests to access these datasets should be directed to yanfeifei2018@163.com.
