# Supplementary material for: Perceived value and educational implications of traditional Chinese medicine nursing: a mixed-methods study
Source: Front Public Health. 2026 Feb 20;14:1763732. doi: 10.3389/fpubh.2026.1763732 (PMC12963323; doi:10.3389/fpubh.2026.1763732)
Supplement: Supplementary file 1 [file Table_1.DOCX]

# Appendix A

**Questionnaire**

**Part 1: General Demographics**

1. Gender:

A. Male

B. Female

2. University:

3. Current Academic Year:

A. Freshman

B. Sophomore

C. Junior

D. Senior

**Part 2: TCM Cultural Background**

4. Which of the following courses have you taken? (Multiple Choice)

A. TCM Nursing

B. TCM

C. Pharmacology of TCM

D. Basic Theories of TCM

E. Diagnostic Studies in TCM

F. Others

5. Through which channels do you acquire TCM nursing knowledge? (Multiple Choice)

A. Books and newspapers

B. Television

C. The Internet

D. School

E. Expert consultation

6. Methods for reviewing classroom knowledge: (Multiple Choice)

A. Doing practice problems

B. Watching online courses

C. Reviewing books and notes

D. Discussing with classmates

E. Seldom review

7. Have you understood the content of “China Citizen Science Literacy Benchmark”?

A. Yes

B. No

8. Have you ever attended a lecture about TCM nursing?

A. Yes

B. No

9. Have lectures on TCM nursing been held in your school?

A. Yes

B. No

10. Did you read books about TCM nursing?

A. Yes

B. No

11. Do any of your relatives work in the field of TCM nursing?

A. Yes

B. No

**Part 3: Cognition of TCM Nursing**

12. Are you willing to learn about TCM nursing?

A. Yes

B. No

13. What is your opinion on the similarities and differences between TCM and Western medicine?

A. They have commonalities as well as their own characteristics.

B. The two are completely different.

14. Do you believe TCM nursing has a positive impact on social healthcare?

A. Yes

B. No

15. What is your view on TCM nursing’s impact on global healthcare?

A. Has a profound impact

B. Has some impact but not significant

C. Others

16. Do you think TCM nursing can effectively treat diseases?

A. Yes

B. No

17. Do you agree that TCM culture has promoted the global spread of Chinese culture?

A. Yes

B. No

18. Do you agree with the concept of TCM nursing treatment?

A. Yes

B. No

19. Do you recognize and approve of TCM nursing?

A. Yes

B. No

20. Do you agree with the establishment of TCM nursing courses in colleges?

A. Agree

B. Disagree

21. How should TCM nursing absorb Western medical nursing?

A. Fully absorb

B. Absorb and draw on according to actual conditions

C. No need to absorb and draw on

**Part 4: Knowledge of TCM Nursing**

22. Which of the “Four Great Classic Works” of TCM are you familiar with? (Multiple Choice)

A. Huangdi Neijing

B. Nan Jing

C. Shennong Bencao Jing

D. Shanghan Zabing Lun

23. Which TCM theories have you learned? (Multiple Choice)

A. Meridian Theory

B. Five Elements Theory

C. Yin-Yang Theory

D. Visceral Manifestation Theory

E. Etiology Theory

24. What do you believe are the core spirits of TCM culture? (Multiple Choice)

A. Unity of Heaven and Man, Harmony and Coexistence

B. Image Thinking, Intuitive Thinking

C. Follow the Natural Way, Strive for Balance

D. Prevent Illness Before It Occurs, Emphasize Health Preservation

25. What are the effects of TCM treatment in your opinion? (Multiple Choice)

A. Preventive care before illness

B. Symptom relief

C. Minimal side effects

D. Specialized in chronic disease treatment

E. Unique therapeutic methods

26. Which organs are included in the “Five Viscera” in TCM theory?

A. Lungs, kidneys, heart, stomach, gallbladder

B. Heart, lungs, gallbladder, kidneys, intestines

C. Heart, lung, spleen, liver, kidneys

D. Stomach, liver, gallbladder, heart, lungs

27. Which description of the generative cycle of the Five Elements is correct?

A. Fire generates Earth

B. Fire gives birth to Metal

C. Fire generates Water

D. Fire produces Wood

28. What is the best container for decocting Chinese herbal medicine?

A. Clay pot

B. Aluminum pot

C. Iron pot

29. What effects does TCM nursing include?

A. Health preservation and wellness

B. Treatment of common diseases

C. Treatment of difficult and complicated conditions

30. What is your understanding of the compatibility and actions of Chinese herbal medicine?

A. They achieve a synergistic effect.

B. They may produce severe toxic side effects.

C. Both of the above are correct.

**Part 5: Behavior of Applying TCM Nursing**

31. Which of the following TCM nursing treatments have you received? (Multiple Choice)

A. Prescriptions

B. Acupuncture

C. Tuina

D. Cupping

E. Acupuncture point massage

32. Which TCM health concepts about daily diet do you agree with? (Multiple Choice)

A. Balanced diet

B. Moderation in diet

C. Seasonal diet

D. Syndrome-based meal planning

33. What is your usual mode of seeking medical treatment?

A. Consultation before choosing

B. See a Western medicine doctor firstly

C. See a TCM doctor firstly

34. Have you ever received treatment from TCM nursing?

A. Yes

B. No

35. Which products related to TCM healthcare have you purchased? (Multiple Choice)

A. Auricular therapy patches

B. Gua sha board

C. Moxibustion sticks

D. Nothing

36.Which TCM health methods do you actively adopt? (Multiple Choice)

A. Tai Chi

B. Zhan Zhuang

C. Yi Jin Jing

D. Ba Duan Jin

E. Nothing

37. Do you introduce TCM nursing knowledge to others?

A. Yes

B. No

38. Do you promote and inherit the culture of TCM?

A. Yes

B. No

**Part 6: Awareness of TCM-related Policies**

39. Which of the following national TCM policies are you aware of? (Multiple Choice)

A. TCM Law of the People’s Republic of China

B. The 14th Five-Year Plan for TCM Development

C. Opinions on Strengthening the Work of TCM Talents in the New Era

D. Completely unfamiliar

40. Which TCM nursing treatments are you aware of that are covered by medical insurance? (Multiple Choice)

A. Acupuncture with filiform needles, Scalp acupuncture therapy

B. Cupping and Tuina therapy

C. Moxibustion and steaming or washing therapy

D. Acupoint plastering, injection, and blood-letting therapy

E. I am not aware of any.

# Appendix B

| **Primary Domain** | **Secondary Topic** | **Example Probes for Depth (To be used-flexibly to deepen-discussion)** |
| --- | --- | --- |
| 1. Perceptions of TCM Nursing | 1.1 Could you share your overall views and perceptions of TCM nursing? | 🞄 When you hear the term “TCM nursing”, what is the first keyword that comes to mind? What do you perceive as the most significant difference between TCM and Western nursing?  🞄 In the current healthcare environment, what value do you think TCM nursing has for you? |
| 2. Learning Experiences and Emotional Responses | 2.1 Could you describe your experiences and feelings during the TCM nursing course? Were there any specific events that left a deep impression on you? | 🞄 You mentioned a memorable moment (e.g., during clinical clerkship or lab sessions); could you describe exactly what happened and why it evoked such a strong reaction?  🞄 During the learning process, did you experience any turning points—such as moving from “confusion” to “sudden realization”, or from “initial interest” to “frustration”? What triggered these changes? |
| 3. Changes in Cultural Understanding and Attitudes | 3.1 After completing the course, how have your cognition and understanding of TCM nursing and treatments evolved? | 🞄 You mentioned that you previously viewed TCM as “mystical” but now see it as “scientific”. How did this transition occur?  🞄 What factors led you to genuinely trust the efficacy of TCM nursing?  🞄 After completing this course, do you believe that TCM thinking helps you become a “more holistic nurse”? In what specific areas (e.g., communication, holistic care, or humanistic concern) is this impact most evident? |
| 4. Impact on Personal Life and Growth | 4.1 How has studying TCM nursing impacted your family, life, or academic perspective? | 🞄 Can you provide a specific example of applying TCM knowledge (e.g., acupressure) to help yourself or a family member?  🞄 How has learning TCM changed the way you look at a patient’s holistic health compared to before? |
| 5. Suggestions for Improving TCM Nursing Education | 5.1 What are your specific needs and suggestions for improving the TCM nursing curriculum? | 🞄 You suggested increasing practical sessions. If you were the instructor, how would you balance the ratio of theory to practice to address the issue of “linguistic obscurity”?  🞄 Beyond classroom teaching, what types of resources (e.g., short videos, hospital clerkships, or interdisciplinary seminars) would best help you build confidence in TCM nursing? |

# Appendix C

## Table 1. Background of nursing undergraduates in TCM nursing.

| Variable | All universities  ( *N*=172, % ) | Western-medicine-oriented university  ( *n*=97, % ) | TCM-oriented university  ( *n*=75, % ) | *X^2^* | *p*-value |
| --- | --- | --- | --- | --- | --- |
| The learned classes include |  |  |  | - | - |
| TCM Nursing | 142 (82.6) | 77 (79.4) | 65 (86.7) |  |  |
| TCM | 35 (20.3) | 8 (8.2) | 27 (36.0) |  |  |
| Pharmacology of TCM | 40 (23.3) | 6 (6.2) | 34 (45.3) |  |  |
| Basic Theories of TCM | 79 (45.9) | 13 (13.4) | 66 (88.0) |  |  |
| Diagnostic Studies in TCM | 17 (9.9) | 2 (2.1) | 15 (20.0) |  |  |
| Others | 3 (1.7) | 0 (0.0) | 3 (4.0) |  |  |
| Ways to acquire knowledge of TCM | |  |  | - | - |
| Books and newspapers | 98 (57.0) | 51 (52.6) | 47 (62.7) |  |  |
| Television | 92 (53.3) | 54 (55.7) | 38 (50.7) |  |  |
| The Internet | 134 (77.9) | 73 (75.3) | 61 (81.3) |  |  |
| School | 130 (75.6) | 68 (70.1) | 62 (82.7) |  |  |
| Expert consultation | 47 (27.3) | 26 (26.8) | 21 (28.0) |  |  |
| Methods for reviewing classroom knowledge | |  |  |  |  |
| Doing practice problems | 103 (59.9) | 48 (49.5) | 55 (73.3) | - | - |
| Watching online courses | 108 (62.8) | 55 (56.7) | 53 (70.7) |  |  |
| Reviewing books and notes | 136 (79.1) | 74 (76.3) | 62 (82.7) |  |  |
| Discussing with classmates | 77 (44.8) | 36 (37.1) | 41 (54.7) |  |  |
| Seldom review | 7 (4.1) | 6 (6.2) | 1 (1.3) |  |  |
| Understood the content of “China Citizen Science Literacy Benchmark” | | | | 0.073 | 0.787 |
| Yes | 60 (34.9) | 33 (34.0) | 27 (36.0) |  |  |
| No | 112 (65.1) | 64 (66.0) | 48 (64.0) |  |  |
| Attended a lecture about TCM | |  |  | 0.787 | 0.377 |
| Yes | 113 (65.7) | 61 (62.9) | 52 (69.3) |  |  |
| No | 59 (34.3) | 36 (37.1) | 23 (30.7) |  |  |
| Lectures held on TCM culture in school | |  |  | 36.174 | <0.001 |
| Yes | 108 (62.8) | 42 (43.3) | 66 (88.0) |  |  |
| No | 64 (37.2) | 55 (56.7) | 9 (12.0) |  |  |
| Read books about TCM |  |  |  | 14.315 | <0.001 |
| Yes | 121 (70.3) | 57 (58.8) | 64 (85.3) |  |  |
| No | 51 (29.7) | 40 (41.2) | 11 (14.7) |  |  |
| Relatives work in the field of TCM | |  |  | 0.309 | 0.578 |
| Yes | 58 (33.7) | 31 (32.0) | 27 (36.0) |  |  |
| No | 114 (66.3) | 66 (68.0) | 48 (64.0) |  |  |

## Table 2. Attitudes and cognition of nursing undergraduates toward TCM nursing.

| Variable | All universities  ( *N*=172, % ) | Western-medicine-oriented university  ( *n*=97, % ) | TCM-oriented university  ( *n*=75, % ) | | *X^2^* | | *p*-value | |  |
| --- | --- | --- | --- | --- | --- | --- | --- | --- | --- |
| Willing to learn about TCM nursing | |  | |  | | - | | 0.259 | |
| Yes | 161 (93.6) | 89 (91.8) | | 72 (96.0) | |  |  |  |  |
| No | 11 (6.4) | 8 (8.2) | | 3 (4.0) | |  |  |  |  |
| Similarities and differences between TCM and Western medicine | | | | | | 0.669 | | 0.414 | |
| They have commonalities as well as their own characteristics | 155 (90.1) | 89 (91.8) | | 66 (88.0) | |  |  |  |  |
| The two are completely different | 17 (9.9) | 8 (8.2) | | 9 (12.0) | |  |  |  |  |
| TCM nursing’s positive impact on social healthcare | | | |  | | - | | 1.000 | |
| Yes | 166 (96.5) | 94 (96.9) | | 72 (96.0) | |  |  |  |  |
| No | 6 (3.5) | 3 (3.1) | | 3 (4.0) | |  |  |  |  |
| TCM nursing’s impact on global healthcare | |  | |  | | 0.002 | | 1.000 | |
| Has a profound impact | 149 (86.6) | 84 (86.6) | | 65 (86.7) | |  |  |  |  |
| Has some impact but not significant | 16 (9.3) | 9 (9.3) | | 7 (9.3) | |  |  |  |  |
| Others | 7 (4.1) | 4 (4.1) | | 3 (4.0) | |  |  |  |  |
| TCM nursing can effectively treat diseases | | | |  | | 2.048 | | 0.152 | |
| Yes | 154 (89.5) | 84 (86.6) | | 70 (93.3) | |  |  |  |  |
| No | 18 (10.5) | 13 (13.4) | | 5 (6.7) | |  |  |  |  |
| TCM culture has promoted the global spread of Chinese culture | | | |  | | - | | 0.730 | |
| Yes | 164 (95.3) | 93 (95.9) | | 71 (94.7) | |  |  |  |  |
| No | 8 (4.7) | 4 (4.1) | | 4 (5.3) | |  |  |  |  |
| Agree with the concept of TCM nursing treatment | | | |  | | - | | 1.000 | |
| Yes | 167 (97.1) | 94 (96.9) | | 73 (97.3) | |  |  |  |  |
| No | 5 (2.9) | 3 (3.1) | | 2 (2.7) | |  |  |  |  |
| Recognize and approve of TCM nursing | |  | |  | | - | | 0.468 | |
| Yes | 164 (95.3) | 91 (93.8) | | 73 (97.3) | |  |  |  |  |
| No | 8 (4.7) | 6 (6.2) | | 2 (2.7) | |  |  |  |  |
| Establishment of TCM nursing courses in colleges | | | |  | | 1.816 | | 0.178 | |
| Agree | 160 (93.0) | 88 (90.7) | | 72 (96.0) | |  |  |  |  |
| Disagree | 12 (7.0) | 9 (9.3) | | 3 (4.0) | |  |  |  |  |
| TCM nursing absorbs on Western medical nursing | | | |  | | - | | 0.899 | |
| Fully absorb | 4 (2.3) | 2 (2.1) | | 2 (2.7) | |  |  |  |  |
| Absorb and draw on according to actual conditions | 160 (93.0) | 91 (93.8) | | 69 (92.0) | |  |  |  |  |
| No need to absorb and draw on | 8 (4.7) | 4 (4.1) | | 4 （5.3） | |  |  |  |  |

## Table 3. Attitudes of nursing undergraduates toward TCM nursing.

| Variable | All universities  (*N*=172, % ) | Western-medicine-oriented university  ( *n*=97, % ) | TCM-oriented university  ( *n*=75, % ) | *X^2^* | *p*-value |
| --- | --- | --- | --- | --- | --- |
| The four great classic works of TCM | |  |  | - | - |
| “Huangdi Neijing” | 163 (94.8) | 95 (97.9) | 68 (90.7) |  |  |
| “Nan Jing” | 65 (37.8) | 45 (46.4) | 20 (26.7) |  |  |
| “Bencao Gangmu” | 124 (72.1) | 66 (68.0) | 58 (77.3) |  |  |
| “Shennong Bencao Jing” | 154 (89.5) | 88 (90.7) | 66 (88.0) |  |  |
| “Shanghan Zabing Lun” | 144 (83.7) | 82 (84.5) | 62 (82.7) |  |  |
| The learned TCM theory |  |  |  | - | - |
| Meridian Theory | 144 (83.7) | 78 (80.4) | 66 (88.0) |  |  |
| Five Elements Theory | 148 (86.0) | 83 (85.6) | 65 (86.7) |  |  |
| Yin-Yang Theory | 156 (90.7) | 84 (86.6) | 72 (96.0) |  |  |
| Visceral Manifestation Theory | 129 (75.0) | 65 (67.0) | 64 (85.3) |  |  |
| Etiology Theory | 110 (63.9) | 54 (55.7) | 56 (74.7) |  |  |
| The core spirits of TCM culture | |  |  | - | - |
| Unity of Heaven and Man, Harmony and Coexistence | 153 (89.0) | 89 (91.8) | 64 (85.3) |  |  |
| Image Thinking, Intuitive Thinking | 84 (48.8) | 41 (42.3) | 43 (57.3) |  |  |
| Follow the Natural Way, Strive for Balance | 125 (72.7) | 72 (74.2) | 53 (70.7) |  |  |
| Prevent Illness Before It Occurs, Emphasize Health Preservation | 131 (76.2) | 70 (72.2) | 61 (81.3) |  |  |
| The effects of TCM treatment |  |  |  | - | - |
| Preventive care before illness | 145 (84.3) | 82 (84.5) | 63 (84.0) |  |  |
| Symptom relief | 130 (75.6) | 71 (73.2) | 59 (78.7) |  |  |
| Minimal side effects | 124 (72.1) | 70 (72.2) | 54 (72.0) |  |  |
| Specialized in chronic disease treatment | 132 (76.7) | 72 (74.2) | 60 (80.0) |  |  |
| Unique therapeutic methods | 124 (72.1) | 66 (68.0) | 58 (77.3) |  |  |
| The five viscera in TCM theory include | |  |  | 0.838 | 0.360 |
| Heart, Lung, Spleen, Liver, Kidneys | 147 (85.5) | 85 (87.6) | 62 (82.7) |  |  |
| Others | 25 (14.5) | 12 (12.4) | 13 (17.3) |  |  |
| The generative cycle of the Five Elements | |  |  | 11.209 | 0.011 |
| Fire generates Earth | 111 (64.5) | 72 (74.2) | 39 (52.0) |  |  |
| Fire gives birth to Metal | 43 (25.0) | 18 (18.6) | 25 (33.3) |  |  |
| Fire generates Water | 9 (5.2) | 5 (5.2) | 4 (5.3) |  |  |
| Fire produces Wood | 9 (5.2) | 2 (2.1) | 7 (9.3) |  |  |
| The best container for decocting Chinese herbal medicine | | |  | 1.138 | 0.286 |
| Clay pot | 160 (93.0) | 92 (94.8) | 68 (90.7) |  |  |
| Others | 12 (7) | 5 (5.2) | 7 (9.3) |  |  |
| The effects of TCM nursing include | |  |  | 7.030 | 0.030 |
| Health preservation and wellness | 90 (52.3) | 59 (60.8) | 31 (41.3) |  |  |
| Treatment of common diseases | 25 (14.5) | 10 (10.3) | 15 (20.0) |  |  |
| Treatment of difficult and complicated conditions | 57 (33.1) | 28 (28.3) | 29 (38.7) |  |  |
| The compatibility and actions of Chinese herbal medicine | | |  | 5.820 | 0.054 |
| Achieve a synergistic effect | 34 (19.8) | 16 (16.5) | 18 (24.0) |  |  |
| May produce severe toxic side effects | 17 (9.9) | 6 (6.2) | 11 (14.7) |  |  |
| Both of the above is correct | 121 (70.3) | 75 (77.3) | 46 (61.3) |  |  |

## Table 4. Nursing undergraduates’ application of TCM nursing.

| Variable | All universities  (*N*=172, % ) | Western-medicine-oriented university  ( *n*=97, % ) | TCM-oriented university  ( *n*=75, % ) | *X^2^* | *p*-value |
| --- | --- | --- | --- | --- | --- |
| The received TCM treatments |  |  |  | - | - |
| Prescriptions | 103 (59.9) | 59 (60.8) | 44 (58.7) |  |  |
| Acupuncture | 73 (42.4) | 30 (30.9) | 43 (57.3) |  |  |
| Tuina | 80 (46.5) | 34 (35.1) | 46 (61.3) |  |  |
| Cupping | 77 (44.8) | 31 (32.0) | 46 (61.3) |  |  |
| Acupuncture point massage | 87 (50.6) | 37 (38.1) | 50 (66.7) |  |  |
| TCM health concepts about daily diet | |  |  | - | - |
| Balanced diet | 143 (83.1) | 81 (83.5) | 62 (82.7) |  |  |
| Moderation in diet | 147 (85.5) | 79 (81.4) | 68 (90.7) |  |  |
| Seasonal diet | 148 (86.0) | 81 (83.5) | 67 (89.3) |  |  |
| Syndrome-based meal planning | 130 (75.6) | 70 (72.2) | 60 (80.0) |  |  |
| Modes of seeking medical treatment | |  |  | 18.189 | <0.001 |
| Consultation before choosing | 100 (58.1) | 54 (55.7) | 46 (61.3) |  |  |
| See a Western medicine doctor firstly | 50 (29.1) | 38 (39.2) | 12 (16.0) |  |  |
| See a TCM doctor firstly | 22 (12.8) | 5 (5.2) | 17 (22.7) |  |  |
| Received treatment from TCM |  |  |  | 1.218 | 0.270 |
| Yes | 133 (77.3) | 72 (74.2) | 61 (81.3) |  |  |
| No | 39 (22.7) | 25 (25.8) | 14 (18.7) |  |  |
| Purchased products related to TCM health care | |  |  | 40.165 | <0.001 |
| Auricular therapy patches | 44 (25.6) | 14 (14.4) | 30 (40.5) |  |  |
| Gua sha board | 33 (19.2) | 12 (12.4) | 21 (28.4) |  |  |
| Moxibustion sticks | 39 (22.7) | 22 (22.7) | 17 (23.0) |  |  |
| Nothing | 56 (32.6) | 49 (50.5) | 6 (8.1) |  |  |
| The actively adopted TCM health methods | |  |  | - | 0.001 |
| Tai Chi | 39 (22.7) | 26 (26.8) | 13 (17.3) |  |  |
| Zhan Zhuang | 11 (6.4) | 7 (7.2) | 4 (5.3) |  |  |
| Yi Jin Jing | 2 (1.2) | 2 (2.1) | 0 (0.0) |  |  |
| Ba Duan Jin | 82 (47.7) | 33 (34.0) | 49 (65.3) |  |  |
| Nothing | 38 (22.1) | 29 (29.9) | 9 (12.0) |  |  |
| Introduce TCM knowledge to others | |  |  | 0.294 | 0.587 |
| Yes | 139 (80.8) | 77 (79.4) | 62 (82.7) |  |  |
| No | 33 (19.2) | 20 (20.6) | 13 (17.3) |  |  |
| Promote and inherit the culture of TCM | |  |  | - | 0.406 |
| Yes | 166 (96.5) | 95 (97.9) | 71 (94.7) |  |  |
| No | 6 (3.5) | 2 (2.1) | 4 (5.3) |  |  |

## Table 5. Nursing undergraduates’ understanding of traditional Chinese medicine policies.

| Variable | All universities  (*N*=172, % ) | Western-medicine-oriented university  ( *n*=97, % ) | TCM-oriented university  ( *n*=75, % ) |
| --- | --- | --- | --- |
| Aware policies about TCM by the country | |  |  |
| TCM Law of the People’s Republic of China | 92 (53.3) | 50 (51.5) | 42 (56.0) |
| The 14th Five-Year Plan for TCM Development | 94 (54.7) | 45 (46.4) | 49 (65.3) |
| Opinions on Strengthening the Work of TCM Talents in the New Era | 69 (40.1) | 30 (30.9) | 39 (52.0) |
| Completely unfamiliar | 43 (25.0) | 31 (32.0) | 12 (16.0) |
| The TCM treatments covered by medical insurance | |  |  |
| Acupuncture with filiform needles, Scalp acupuncture therapy | 80 (46.5) | 39 (40.2) | 41 (54.7) |
| Cupping and Tuina therapy | 75 (43.6) | 34 (35.0) | 41 (54.7) |
| Moxibustion and steaming or washing therapy | 67 (39.0) | 29 (29.9) | 38 (50.7) |
| Acupoint plastering, injection, and blood-letting therapy | 71 (41.3) | 29 (29.9) | 42 (56.0) |
| Be not aware of it | 67 (39.0) | 47 (48.4) | 20 (26.7) |
